# Influence of colloidal nano silica on solidification mechanisms and hydration process of nano modified cement

**DOI:** 10.1038/s41598-025-24840-9

**Published:** 2025-11-12

**Authors:** Asmaa Nouh, Hoda Abou-Shady, Rehab O. Abdel Rahman

**Affiliations:** 1 Physics Department, Faculty of Science, Cairo University, Giza, Egypt; 2 Hot Laboratory Center, Egyptian Atomic Energy Authority, Inshas, Cairo, Egypt

**Keywords:** Nano-modified cement, Early age behavior, Mathematical models, Setting time, Mechanical strength, Hydration mechanisms, Chemistry, Engineering, Environmental sciences, Materials science, Nanoscience and technology

## Abstract

This study assesses the feasibility of using Colloidal Nano-Silica (CNS) as a modifier for the radioactive waste immobilization matrix by investigating its effects on the setting time, soundness, mechanical stability, microstructure evolution, and solidification mechanism in simplified cementitious systems. It provides highlights into the isolated role of the CNS in the evolution of the solidification process, analyzes the temporal sensitivity of the mechanical strength evolution and its dependency on the CNS dosage, and provides mechanistic insights into the role of CNS in the changes in the controlling hydration reaction. In this respect, the nano-silica was synthesized via the sol–gel method and extensively characterized to determine its physicochemical properties. The resulting amorphous material, with a particle size below 2.49 nm and approximately 20 wt.% of weakly bound and chemically adsorbed water, forms a stable colloidal solution with a zeta potential of -33.9 mV. Incorporating CNS into cementitious matrices notably altered hydration kinetics, mechanical performance. and improves the durability. CNS accelerated both initial and final setting, exhibiting a non-monotonic trend as a function of CNS content, attributed to its water adsorption capacity, most pronounced at 5 wt.%. All formulations maintained acceptable soundness, with optimal enhancement observed at 3 wt.%, beyond which minor deterioration was detected at 5 wt.%. Although compressive strength generally declined with CNS addition over the curing period, the values remained above the minimum threshold for use in radioactive waste backfilling and waste immobilization. Mathematical analysis of the behavior over a 45-day curing period revealed that compressive strength was relatively insensitive to CNS variation, particularly between 1.5% and 3% material incooperation. Temporal analysis further indicated that strength development was limited during early curing stages but became material-specific at later ages. The improved durability of the 3% CNS-supplemented materials is related to the lime-silica reaction and the formation of C-S–H of low ca/si ratio. Mechanistic insights suggest that CNS addition promotes nucleation and growth mechanisms at the expense of diffusion-driven hydration, with critical transitions observed at 3% and 5% CNS content.

## Introduction

Cementitious materials are integral to the nuclear industry, forming a foundational element in numerous structures, systems, and components (SSCs) that support essential operational and safety functions. These include ensuring structural integrity, providing radiation shielding, and contributing to radiological containment and isolation^1^. Due to their passive nature, cement-based SSCs are widely applied in both active systems, such as those found in nuclear reactors, and in facilities designed for passive safety, particularly those dedicated to radioactive waste disposal^2^.

Within disposal facilities, these materials serve not only as structural components but also as waste forms and engineered backfill in several disposal concepts, including both near-surface and geological facilities. Additionally, they are proposed for their application in geological disposal sealing. Their safety classifications and functions vary according to the design objectives of the disposal system^1^. But generally, they should be designed to ensure the availability of the containment and isolation safety functions in these facilities over an extended time^1^. This sets stringent requirements on the long-term behavior of their sealing properties, mechanical stability, chemical durability, and resistance to radiation-induced degradation^1,3–6^.

Although Ordinary Portland Cement (OPC) continues to dominate these SSC’s formulations, recently an increased interest in the application of alternative cementitious systems, including calcium sulfoaluminate, calcium aluminate, magnesium phosphate, and geopolymer-based cements^7–9^, the immobilizing of challenging waste streams is noted. However, the industrial adoption of these alternatives remains limited to countable applications in low and intermediate radioactive waste immobilization, with no proposed applications in other disposal SSCs^10–15^. The limited long-term understanding and operational data of these systems and their limited standards and regulations are key challenges for their wide applications^7,12,16–19^. The immaturity of these alternative systems motivates researchers to enhance the OPC system’s performance rather than replacing it. This enhancement can be achieved by relying on the incorporation of supplementary materials to ensure their compliance with the above-mentioned stringent requirements on the performance of OPC-based SSCs.

To ensure long-term performance, understanding the evolution of the cement solidification process is essential for these safety–critical SSCs. In particular, attention has turned to the role of supplementary materials in enhancing their solidification behavior. Both organic and inorganic additives have been explored for their potential to improve the mechanical performance and durability^20,21^. Among these, pozzolanic nanomaterials such as nano-silica, metakaolin, and fly ash have attracted considerable attention due to their high surface area and chemical reactivity^22–24^.

These materials are typically assessed within composite cementitious matrices containing OPC and aggregates, with or without chemical accelerators. Yet to isolate the role of nano-additives, simpler systems comprised solely of OPC and a single nano-supplementary material provide a more direct understanding. Such systems are relevant to specific applications in waste immobilization and engineered backfill in disposal settings^15,25–27^.

In this respect, limited researches have addressed the effect of incorporating zeolite and nano-silica in cementitious radioactive waste form, to improve the solidification and durability performances of innovative cementitious materials, i.e., Sulfoaluminate cement^28,29^. Additionally, limited published works focused on the application of ferrihydrite nano-particles to enhance the immobilization of iodine in radioactive waste forms^30,31^. Another research revealed that the use of micro- and nano-silica enhanced the performance of OPC concrete waste form^32^. Additionally, several researches addressed the role of nano silica on the thermal performance, forest resistance, and pore structure^33,34^. Chen et al. (2024) concluded that despite nano-materials have the potential to improve the performance of the cementitious waste forms, yet their application is limited by the dispersion of these materials in the cementitious waste forms^33^. Hence, their practical implementation still requires investigations to improve their dispersion and fully understand their roles in the solidification process^10,17,35^.

In general, silica-based supplementary materials are proposed to improve the mechanical strength and reduce the porosity^32–36^. Additionally, it can increase the pH of the hardened materials, leading to a better radionuclide containment performance and improving the corrosion resistance of the waste packages. Despite these advantages, yet their role in the solidification process is not clearly addressed. In particular, the detailed analysis of the effect of the incorporation of nano-silica on the solidification process and its effect on the evolution of the pore structure of the cementitious system is still poorly explored, with no clear dosage optimization based on mechanistic insights into their role in the solidification process.

The present study investigates the role of colloidal nano-silica in the solidification behavior of a simplified OPC-based system to address the poor dispersion of nano-silica in this system. In this respect, nano-silica was synthesized via the sol–gel method and characterized using a range of physicochemical techniques. Cement pastes were prepared with varying nano-silica contents, and the progression of the solidification process was assessed via setting time, soundness, strength activity index, and compressive strength. Mathematical models were developed to interpret the evolution of pore structure and to provide mechanistic insight into the cement hydration pathways.

## Materials and methods

### Materials

**Fig. 1:**
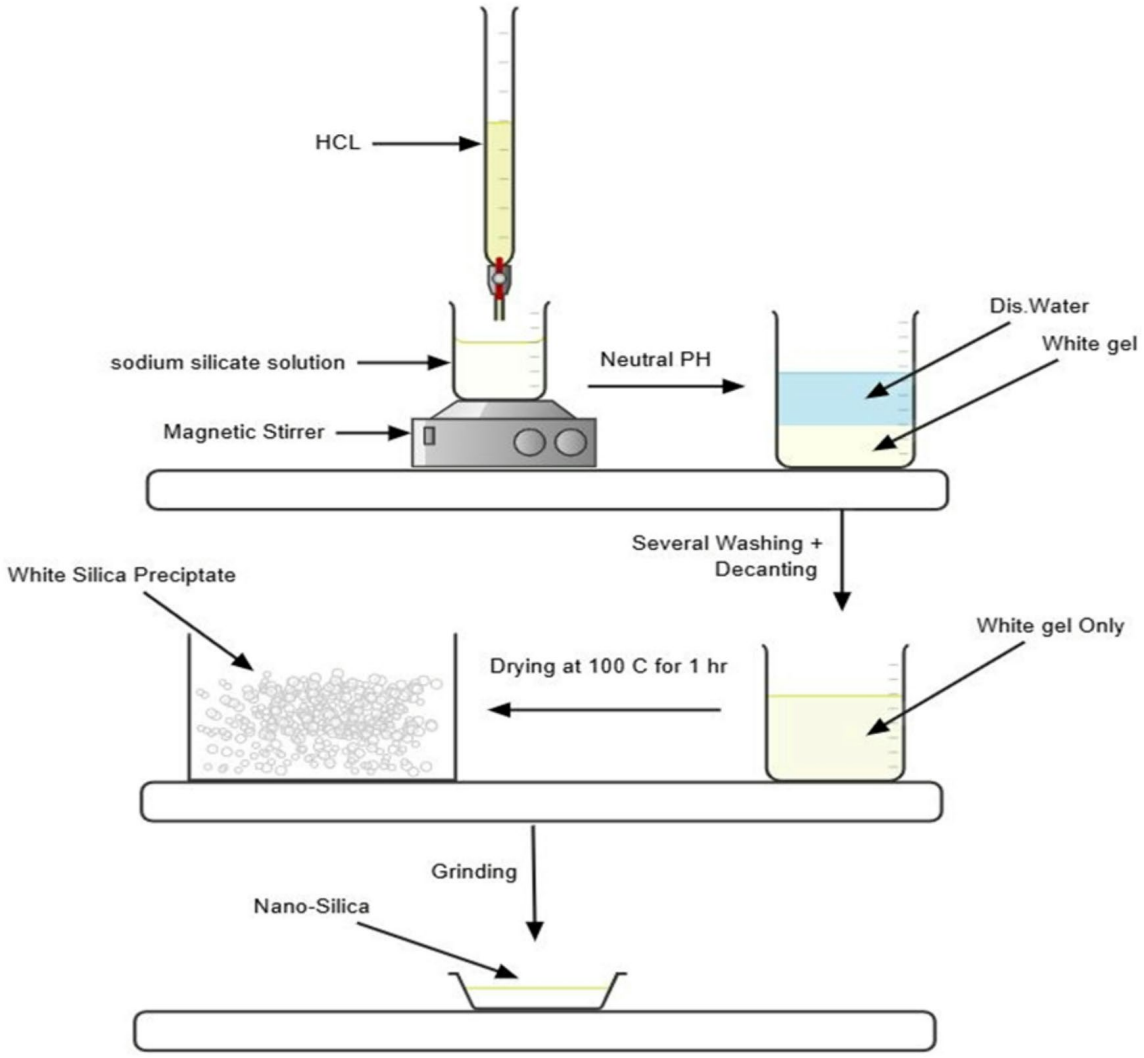
Schematic representation of the Nano-silica preparation using the sol–gel method.

Colloidal nano-silica was synthesized via the sol–gel method (Fig. 1) using analytical-grade sodium silicate solution (50 mL) and hydrochloric acid (2 M). The reaction was conducted at ambient temperature by gradually adding the acid dropwise under continuous stirring until a white gel precipitated and the solution pH reached a neutral range (6.5–7), in accordance with previous protocols^37,38^. The resulting gel was thoroughly washed to eliminate residual ions, dried at 100 °C for one hour, and subsequently milled to achieve nano-scale particle sizes.

Ordinary Portland Cement (OPC; sample S0), rated at 42.5 MPa, was employed to prepare the cementitious specimens and has the following oxide composition: CaO (61.28 wt.%), MgO (2.8 wt.%), SiO₂ (20.46 wt.%), Al₂O₃ (5.14 wt.%), Fe₂O₃ (3.53 wt.%), SO₃ (2.82 wt.%), Na₂O (0.20 wt.%), K₂O (0.11 wt.%), and others (3.66 wt.%). To evaluate the influence of nano-silica on cement solidification, three mix formulations, i.e., S1-S3, were designed as outlined in Table 1. The chemical parameters of each mix, specifically the lime saturation factor (LSF), silica ratio (SR), and calcium-to-silica ratio (Ca/Si), were calculated using standard methods Eq. (1) and Eq. (2)^11,39^. to provide a mechanistic explanation of the obtained setting and expansion behaviors related to the changes in the mix design.

1 $${\text{LSF\% }} = \frac{{{\text{CaO}} + 0.75{\text{MgO}}}}{{2.8{\text{SiO}}_{2} + 1.18{\text{Al}}_{2} {\text{O}}_{3} + 0.65{\text{Fe}}_{2} {\text{O}}_{3} }} \times 100$$

2 $${\text{SR}} = \frac{{{\text{SiO}}_{2} {\text{\% }}}}{{{\text{Al}}_{2} {\text{O}}_{3} + {\text{Fe}}_{2} {\text{O}}_{3} }}$$

**Table 1:** Effect of nano-silica incorporation on the solidification process: mix design at normal consistency.

Samples	S0	S1	S3	S5
Nano-silica, %	0	1	3	5
CaO, %	61.28	63.61	62.97	61.69
MgO, %	2.8	2.91	2.88	2.81
SiO2, %	20.46	21.24	22.02	23.60
Al2O3, %	5.14	5.33	5.28	5.17
Fe2O3, %	3.53	3.66	3.63	3.55
SO3, %	2.82	2.92	2.89	2.83
Na2O, %	0.2	0.21	0.21	0.20
K2O, %	0.11	0.11	0.11	0.11
Others, %	3.66	3.79	3.76	3.68
LSF	96.55	92.7	85.66	79.38
SR	2.36	2.47	2.70	2.94
Ca/Si	2.99	2.86	2.61	2.40

Table 1 shows an increase in the LSF and SR values by increasing the incorporation of the CNS in the mix design, implying its effects on the sample expansion and the acceleration of the solidification process^11^.

## Nano-silica characterization

The synthesized nano-silica was subjected to comprehensive physicochemical characterization. Surface morphology was examined using Atomic Force Microscopy (AFM; Model 5600LS, Agilent Technologies, USA). The chemical structure was analyzed via Fourier Transform Infrared (FTIR) Spectroscopy using the KBr pellet technique, with spectra recorded on a Nicolet 6700 spectrometer (Thermo Scientific, USA). X-ray diffraction (Bruker D8) pattern was collected to confirm the amorphous nature of the material. Nitrogen adsorption–desorption measurements were conducted using a Quanta Chrome Nova-Win instrument to determine the specific surface area and pore volume. Thermogravimetric analysis (TGA) was employed to assess thermal stability. Finally, Zeta potential was measured to evaluate colloidal stability.

## Characterization of the solidification process

Setting times and consistency were determined using Vicat apparatus according to ASTM C191-21. The soundness expansion (SE, mm) is an expression of the dimensional stability of cement paste; it was assessed using the Le Chatelier method, following the BS EN 197-1 standard. It is evaluated by calculating the difference in the sample dimensions before and after immersion in water at 27 °C for 24 h and boiling for 3 h Eq. (3):

3 $${\text{SE}} = {\text{B}} - {\text{A}}$$

where A (mm) is the dimensions before immersion; B (mm) is the dimensions after immersion.

Samples were classified as unsound if SE exceeded 10 mm. The development of compressive strength over time was monitored to assess the influence of nano-silica on the solidification behavior. Measurements were conducted in accordance with ASTM C109. Strength Activity Index (SAI) values were calculated using 50 mm cubic samples, as prescribed by ASTM C311 Eq. (4)^40^.

4 $${\text{SAI}} = { }\frac{{{\upsigma }_{{{\text{SX}}}} }}{{{\upsigma }_{{{\text{S}}0}} }} \times 100$$

The evaluation of the pore structure of cement-based materials using direct methods is challenged on one hand by the wide range of pore sizes and their heterogeneous nature^41^ , and on the other hand, by the uncertainties associated with the pretreatment of the samples and the techniques limitations^42–44^. The porosity of the cement-based materials comprises fine pores (< 10 nm) and coarse pores (10 − 1000 nm). The first are gel pores and they are associated with the formation of the calcium silicate hydrates (C-S–H). The second are capillary pores that are formed due to the water evaporation and they control the transport through the materials, i.e., contaminant transport through the cementitious SSC. To explore the pore structure evolution, i.e., the gel pore volume (${\varphi }_{G}$; Eq. 5), capillary pore volume (${\varphi }_{C}$; Eq. 6), and total porosity (${\varphi }_{T}$; Eq. 7) direct method have been used, i.e., Power’s models^45–48^.

5 $${{\upvarphi }}_{{\text{G}}} = \frac{{0.19\left( {{\raise0.7ex\hbox{${\text{t}}$} \!\mathord{\left/ {\vphantom {{\text{t}} {_{{\text{m}}} }}}\right.\kern-0pt} \!\lower0.7ex\hbox{${_{{\text{m}}} }$}}} \right)}}{{\left( {{\raise0.7ex\hbox{${\text{w}}$} \!\mathord{\left/ {\vphantom {{\text{w}} {\text{c}}}}\right.\kern-0pt} \!\lower0.7ex\hbox{${\text{c}}$}}} \right) + 0.32}}$$

6 $${{\upvarphi }}_{{\text{C}}} = \frac{{\left( {{\raise0.7ex\hbox{${\text{w}}$} \!\mathord{\left/ {\vphantom {{\text{w}} {\text{c}}}}\right.\kern-0pt} \!\lower0.7ex\hbox{${\text{c}}$}}} \right) - 0.36\left( {{\raise0.7ex\hbox{${\text{t}}$} \!\mathord{\left/ {\vphantom {{\text{t}} {_{{\text{m}}} }}}\right.\kern-0pt} \!\lower0.7ex\hbox{${_{{\text{m}}} }$}}} \right)}}{{\left( {{\raise0.7ex\hbox{${\text{w}}$} \!\mathord{\left/ {\vphantom {{\text{w}} {\text{c}}}}\right.\kern-0pt} \!\lower0.7ex\hbox{${\text{c}}$}}} \right) + 0.32}}$$

7 $${{\upvarphi }}_{{\text{T}}} = \frac{{\left( {{\raise0.7ex\hbox{${\text{w}}$} \!\mathord{\left/ {\vphantom {{\text{w}} {\text{c}}}}\right.\kern-0pt} \!\lower0.7ex\hbox{${\text{c}}$}}} \right) - 0.17\left( {{\raise0.7ex\hbox{${\text{t}}$} \!\mathord{\left/ {\vphantom {{\text{t}} {_{{\text{m}}} }}}\right.\kern-0pt} \!\lower0.7ex\hbox{${_{{\text{m}}} }$}}} \right)}}{{\left( {{\raise0.7ex\hbox{${\text{w}}$} \!\mathord{\left/ {\vphantom {{\text{w}} {\text{c}}}}\right.\kern-0pt} \!\lower0.7ex\hbox{${\text{c}}$}}} \right) + 0.32}}$$

where $\sigma_{t}$ (MPa) is the compressive strength measured at a specified time (t), $\sigma_{m}$ (MPa) is the ultimate compressive strength calculated by using Eq. 8.

8 $${\upsigma }_{{\text{m}}} = {\upsigma }_{{\text{t}}} \left( {\frac{{1.031\left( {{\raise0.7ex\hbox{${\text{w}}$} \!\mathord{\left/ {\vphantom {{\text{w}} {\text{c}}}}\right.\kern-0pt} \!\lower0.7ex\hbox{${\text{c}}$}}} \right)}}{{1.94 + \left( {{\raise0.7ex\hbox{${\text{w}}$} \!\mathord{\left/ {\vphantom {{\text{w}} {\text{c}}}}\right.\kern-0pt} \!\lower0.7ex\hbox{${\text{c}}$}}} \right)}} + 0.5{\text{ NS}}} \right)$$

Moreover, the role of CNS on the development of the solidification process was investigated by collecting and analyzing the FTIR and TGA patterns for Blank and 3% CNS-supplemented cementitious samples that has improved setting time and soundness.

## Determination of the solidification mechanism

To elucidate the dominant mechanisms governing the cement hydration process, non-linear regression analysis was applied to experimental strength data using three kinetic models: nucleation and growth (Eq. 9), diffusion-controlled (Eq. 10), and a hybrid chemical reaction/diffusion model (Eq. 11)^11,45,49–52^.

9 $${\upsigma }_{{\text{t}}} = {\upsigma }_{{\text{u}}} \left( {1 - {\text{e}}^{{ - \left( {{\text{k}}_{{{\text{NG}}}} {\text{t}}} \right)^{{\text{n}}} }} } \right)$$

10 $${\upsigma }_{{\text{t}}} = {\upsigma }_{{\text{u}}} \left( {1 - \left( {1 - \sqrt {{\text{k}}_{{\text{d}}} {\text{t}}} } \right)^{{\text{n}}} } \right)$$

11 $${\upsigma }_{{\text{t}}} = {\upsigma }_{{\text{u}}} \left( {1 - \left( {1 - {\text{k}}_{{\text{i}}} {\text{t}}} \right)^{{\text{n}}} } \right)$$

where k_x_ is the rate constant (d^-1^), between the solid/liquid phases and mass transfer through the hydrated phases, and n is the model constant.

## Results and discussion

### Physicochemical characterization of nano-silica

**Fig. 2:**
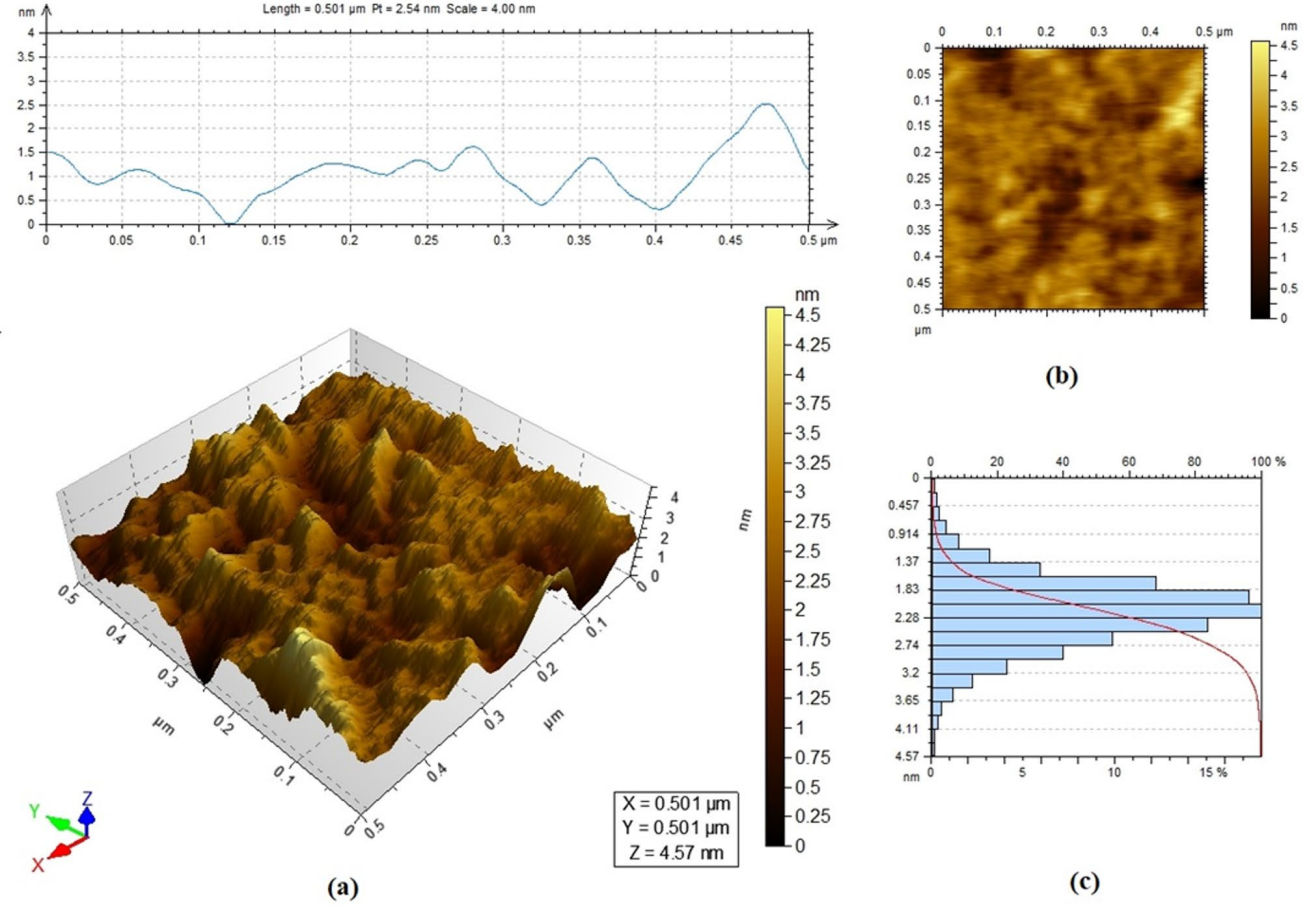
Morphology of the prepared nano-silica (**a**) lateral view with corresponding surface roughness, (**b**) top view, (**c**) histogram of the surface roughness.

Figure 2 displays the morphology and surface characteristics of the prepared nano-silica; the lateral roughness of the surface is varied (Fig. 2a), up to 2.49 nm. The top view of the material (Fig. 2b) shows a fairly flat surface with a void volume equal to 7% and the typical roughness histogram distribution (Fig. 2c) illustrates that the mean surface roughness is about 2 nm. Nano-silica roughness can play an important role in the hydration process, where increased roughness implies larger water absorption on the material surface and a reduction of the available water for hydration. The significance of this effect and its impact on the efficacy of the solidification process is dependent on the nano-material dosage in the cementitious samples and the water-to-cement ratio.

**Fig. 3:**
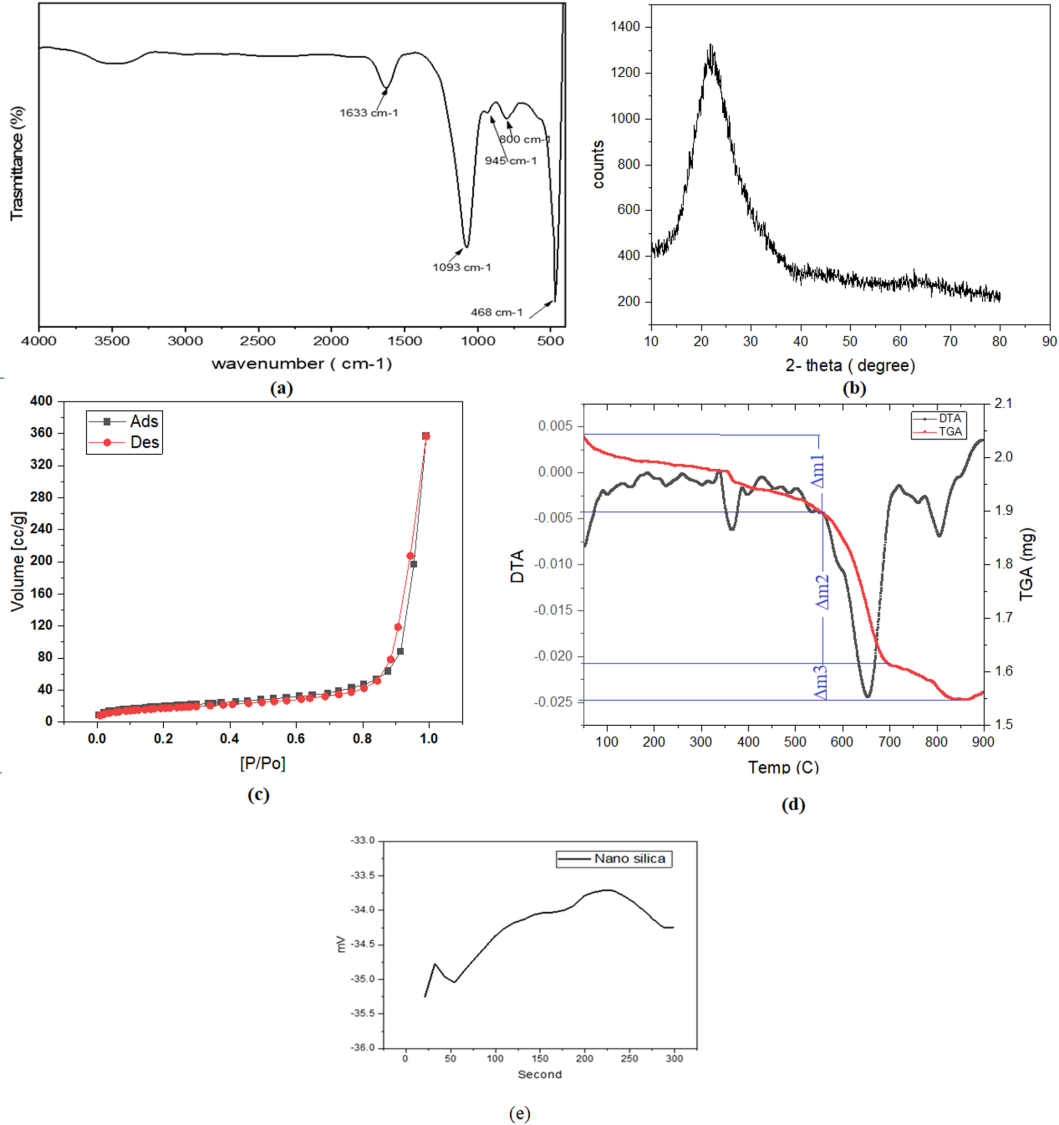
Characterization of the prepared nano-silica, (**a**) mid infrared spectrum, (**b**) XRD pattern, (**c**) N2 adsorption–desorption isotherm, (**d**) thermo gravimetric analysis, (**e**) zeta potential measurement.

Mid-infrared spectrum of nano-silica is illustrated in (Fig. 3a), six distinguished peaks characterize the material and represent the OH stretching and bending vibrations of the silanol or absorbed water at 3440 and 1633 cm⁻^1^, respectively,^37,53,54^. A Predominant band is noted at 1093 cm⁻^1^ , which corresponds to the asymmetric stretching of the Si–O–Si bonds^37,53,55^. Two small peaks are detected at 945 and 800 cm⁻^1^ are related to the stretching vibrations of the Si–O bond that represent amorphous silica and protonated amorphous silica^56^. The band observed at 468 cm⁻^1^ is due to the in-plane Si–O bending vibrations^53,54,56^. (Fig. 3b) shows a broad notable band in the XRD pattern around 2Փ = 22⁰, which confirms the amorphous nature of the sample^57^. The disappearance of the sharp Bragg peaks and the softness of the band also indicate the absence of peaks of possible crystalline impurities^37,57–59^. The nitrogen adsorption–desorption isotherm for the prepared material is illustrated in (Fig. 3c); the specific surface area (SSA) and total pore volume (TPV) are equal to 69.87 m^2^/g and 0.57 cm^3^/g, respectively. In comparison to other silica sources that were studied to supplement the cementitious materials, the prepared nano-silica has higher SSA than silica fume (20 m^2^/g), quartz particles (4 m^2^/g)^60^, and commercial nano silica (33.7 and 45m^2^/g)^61,62^. Thermo-gravimetric data are illustrated in (Fig. 3d). Three distinguishable slopes of the TGA data represent mass losses equal to 6.86, 14.73, and 3.7% in the temperature ranges < 600 ℃, 600–700 ℃, and 700–900 ℃. DTA data show four major endothermic peaks in the ranges < 100, 340–380, 560–700, 760–840 ℃. The first is attributed to the loss of the weakly bounded OH groups on the surface^37,45,63,64^. The second peak can be explained by the loss of the chemically sorbed water bounded to the Si–OH through hydrogen bond; i.e., vicinal hydroxyl groups, whereas the third and fourth peaks represent the desorption of Si–OH groups and removal of structural water, i.e., germinal OH and isolated single groups^63,65^. These peaks are associated with the condensation of silanol groups (Si–OH) to siloxane bonds (Si–O-Si)^65,66^. Finally, the zeta potential of nano-silica is -33.96mV, which reflects that the material remains in colloidal form under sonication for more than 300 s^67^ (Fig. 3e). This value is comparable to the colloidal silica used to supplement cement-based materials^60^.

## Solidification process characterization

**Fig. 4:**
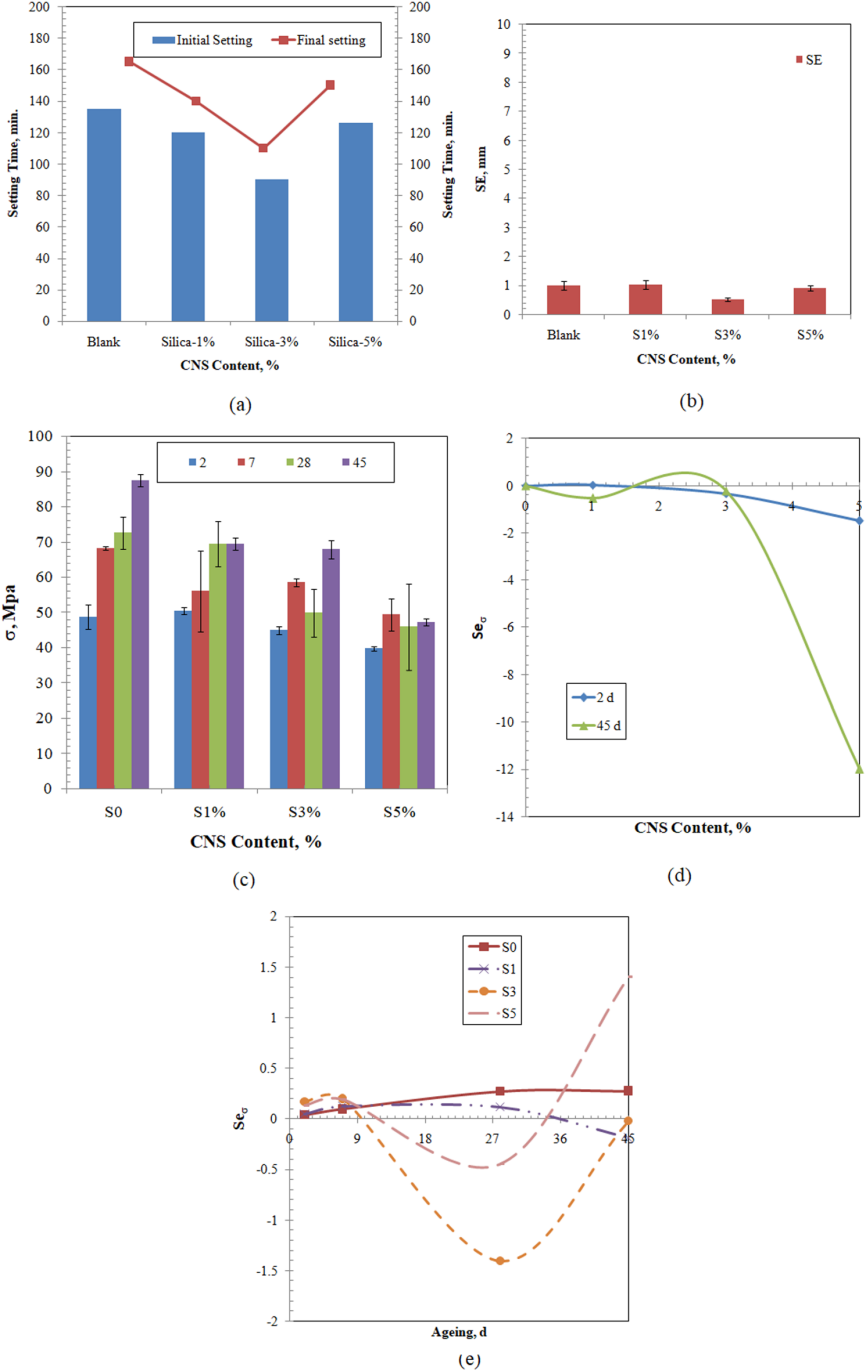
Characterization of the effect of CNS content on the evolution of the solidification process (**a**) setting times, (**b**) soundness at 1 day, (**c**) compressive strength over 45 days, (**d**) normalized sensitivity coefficients (Seσ) of the compressive strength at 2 and 45 days, and (**e**) (Seσ) for the studies samples.

The effect of increasing the incorporation of colloidal nano-silica (CNS) in the cementitious samples during the early age of the solidification process is examined by evaluating the initial and final setting times (Fig. 4a.) Illustrates the reduction of the initial and final setting times due to the progressive increase in the incorporation of the colloidal nano-silica, which can be attributed to increasing the silica ratio. This is in conformance with reported studies that reveal the role of nano-silica in accelerating the hydration process and hence reducing the setting time^62,68^. This effect does not have a monotonic behavior, where the addition of 1% will reduce the initial and final setting times by 11.11% and 15.15%, respectively. Further increase in the amount of colloidal nano-silica to 3% will reduce the setting times by 33.33% for both the initial and final setting times. Finally, the use of 5% will reduce the setting times by 6.67 and 9.01%, which is still less than that of the blank sample. Similar non-monotonic effect of the progressive increase of colloidal nano-silica in the cement samples was reported for the use of nano-silica to supplement high sulfate resistance Portland cement^68^. This behavior can be explained by the availability of a larger surface area to adsorb the water; hence, the amount of available water to react with the cement will be reduced^68,69^. The blank sample shows soundness equals 1 mm after 1 day, the addition of 1% colloidal nano-silica did not affect the soundness largely, whereas the addition of 3 and 5 % reduced the soundness of the samples (Fig. 4b). This result revealed that all the samples are fairly sound, i.e., <10 mm. The effect of silica on the sample expansion can be explained by its reaction with the free lime, that is reduced by increasing the silica incorporation (Table 1), to form amorphous C-S-H, with high calcium to silica ratio, and hence inhibits its hydration^70^. It should be noted that this behavior is dependent on the amount of the supplemented silica, where the addition of 40% of silica was reported to eliminate the expansion of the cementitious samples containing high calcium fly ash^70,71^. The increase of the amount of colloidal nano-silica in the cementitious sample to 5% is associated with a slight increase in the sample expansion compared to that of the 3% but it is still less than that of the blank sample. This can also be attributed to the availability of the surface to absorb the water and hence reduce the water available for the hydration reaction.

The effect of the amount of colloidal nano-silica on the evolution of the solidification process was further investigated in terms of the value of the compressive strength over 45 days as illustrated in Fig. 4c, the incorporation of the colloidal nano-silica led to a decrease in the compressive strength of the solidified samples over the examined curing time, yet this value is higher than that required for the cementitious materials used in the solidification of the radioactive waste and as backfill of disposal facilities(< 10 MPa)^64^. At 2 days, the blank sample has a compressive strength of 48.77±3.53MPa; whereas this value increases to 50.43±0.94 MPa with the addition of 1% of the colloidal nano-silica. Further increase in the content of the colloidal nano-silica will reduce the compressive strength gradually to 44.85 ± 1.04 and 39.65 ± 0.66 MPa for samples S3 and S5, respectively. This behavior may be attributed to the reduction of the available water to undergo the hydration reaction due to its sorption on the colloidal nano-silica surface. At 45 days, this effect is becoming more obvious, where the blank sample has a compressive strength of 87.4 ± 1.69 MPa; and the supplemented samples have compressive strengths equal to 69.45 ± 6.3, 49.85 ± 6.82, and 45.99 ± 12.22 MPa, for 1%, 3%, and 5% colloidal nano-silica content, respectively. In this context, it is worth noting that the effect of the nano-silica incorporation on the compressive strength of the cementitious materials is highly dependent on the studied base cement material, the presence of other supplementary materials, the size of the nano-silica, the dispersion of the nano-silica, and the dose of the nano-silica^55,67,72–77^. The experimental data were analyzed mathematically to investigate the dependency of the compressive strength at 2 and 45 days on the amount of colloidal nano-silica. Strong quadratic and cubic dependencies were found between the compressive strength (σ, MPa) at days 2 and 45, respectively, and the amount of colloidal nano-silica (CNS, %); as follows:

12 $${\upsigma }_{2} = - 1.72{\text{CNS}}^{2} + { }5.29{\text{CNS}} + 45.56,\;\;{\text{R}}^{2} = 0.957$$

13 $${\upsigma }_{45} = - 5.88{\text{CNS}}^{3} + 43.48{\text{CNS}}^{2} - 107.2{\text{CNS}} + 157,\;\;{\text{R}}^{2} = 1$$

This change in the nature of the dependency of the compressive strength on the amount of CNS is related to the change in the chemical composition of the hydrated cementitious material with the progress of the hydration reaction with time. At 2 days, the compressive strength is having a local maximum at 1.54% colloidal nano-silica incorporation (Eq. 12). At longer curing time, i.e. 45 days, the compressive strength is having an inflection point at 2.46% of colloidal nano-silica incorporation (Eq. 13). The sensitivity of the compressive strength to the changes in the content of colloidal nano-silica was determined by calculating the normalized sensitivity coefficient(Se_σ_; Eq. 14)^53,78–80^.

14 $${\text{Se}}_{{{\sigma x}}} = {\text{S}}_{{{\sigma x}}} \left. {\frac{{{\text{CNS}}}}{{\upsigma }}} \right|_{{\text{O}}}$$

where S_σx_ is the direct sensitivity coefficient at time x and equals the first derivative of the dependency equation with respect to the colloidal nano-silica content; hence, the normalized sensitivity coefficients for the compressive strength at days 2 and 45 are given in Eqs. 15 and 16, respectively.

15 $${\text{Se}}_{{{\upsigma }_{2} }} = \left( { - 3.44{\text{CNS}} + 5.29} \right)\left. {\frac{{{\text{CNS}}}}{{\upsigma }}} \right|_{{\text{O}}}$$

16 $${\text{Se}}_{{{\upsigma }_{45} }} = { }\left( { - 17.64{\text{CNS}}^{2} + 86.96{\text{CNS}} - 107.2} \right)\left. {\frac{{{\text{CNS}}}}{{\upsigma }}} \right|_{{\text{O}}}$$

Figure 4d shows that the compressive strength value after 2 days is less sensitive to the change in the amount of the incorporated CNS in the sample compared to that occurring at 45 days. At 45 days, the value of the compressive strength is less sensitive to the CNS incorporation at low doses, i.e., CNS ≤ 3%, where in the range 1.5-3% the normalized sensitivity coefficient has positive values, indicating the positive impact of the CNS on the compressive strength in this range. Finally, the dependency of compressive strength for each sample on the aging time was analyzed mathematically. The compressive strength was found to have the following dependencies (Eqs. 17–20):

17 $${\upsigma }_{{{\text{blank}}}} = - 0.005{\text{t}}^{2} + 0.983{\text{t}} + 52.95,\;\;{\text{R}}^{2} = 0.833$$

18 $${\upsigma }_{{1{\text{\% }}}} = - 0.017{\text{t}}^{2} + 1.24{\text{t}} + 48.08,\;\;{\text{R}}^{2} = 0.999$$

19 $${\upsigma }_{{3{\text{\% }}}} = 0.003{\text{t}}^{3} - 0.256{\text{t}}^{2} + 4.78{\text{t}} + 36.29,\;\;{\text{R}}^{2} = 1$$

20 $${\upsigma }_{{5{\text{\% }}}} = 0.002{\text{t}}^{3} - 0.154{\text{t}}^{2} + 3.188{\text{t}} + 33.88,\;\;{\text{R}}^{2} = 1$$

The normalized sensitivity coefficients of the compressive strength of the studied samples with respect to the ageing time were calculated and plotted as indicated in (Fig. 4e). It is clear from that figure that all the compressive strengths of all the samples are respectively insensitive to the changes in the time within the first week, where the normalized sensitivity coefficient is in the range 0.03 ≤ Se_σ_ ≤ 0.19. This reveals that the development of the compressive strength at this time is fairly constant, where the developed strength is dependent on the main hydration products of the C_3_S and C_2_S with water. A small increase in the normalized sensitivity of the compressive strength is noted for the supplemented samples with the CNS. This increase in the values of the normalized sensitivity coefficients can be attributed to the reaction of the nano-silica with the free lime as revealed from the Le Chatelier test, where the reaction products increase slightly the strength with increasing CNS in the samples^81^. As time increases, the sensitivity of the strength of the samples becomes material-specific. For the blank sample, positive normalized sensitivity coefficients are slightly increased with time, indicating that the compressive strength is less sensitive to the changes in the ageing time. The sample that contains 1% of the CNS has similar normalized sensitivity coefficient behavior to that of the blank sample up to 28 days. At 45 days, the value of the normalized sensitivity coefficient reduced greatly to a negative value, which might refer to the loss of strength development at that age. Further increase in the content of the colloidal nano-silica is associated with a loss of strength at day 28. Similar losses of early strength were reported for the reaction of nano-silica, with particle size 100nm, at 5% dosage, with Portlandite^81^. The reduced strength at 28 days can be explained by the shrinkage associated with the presence of nano-silica^82–86^. The increase in normalized sensitivity coefficient values at 45 days might be related to the changes that occur in the material due to the pozzolanic activity of the CNS. To have insights into the pozzolanic reactivity of the samples, the strength activity indices (SAI) were calculated for the samples using Eq. 4 at ages 2 and 45 days, and the results are listed in Table 2. An increase is noted for the SAI values during the progress of the solidification process for all the modified samples. Similar results were reported for the SAI of cementitious composite, i.e., cement/sand material, with 3% CNS at W/C ratio = 0.5^86^. SAI values at age 2 days are reducing with increasing CNS incorporation, yet the lowest value at 5% CNS incorporation is higher than the threshold value of 75% for supplemented cements^87^. A similar trend is noted for the effect of the increased incorporation of CNS in the solidified samples at 45 days, which implies that the pozzolanic reactivity of the material is increased during this period. This confirms that the reaction between CaO and CNS takes place at both the early age and at longer times of the reaction. Hence, it could be concluded that the reaction of CNS with lime at early (2 days) and longer (45 days) times is responsible for increasing the normalized sensitivity coefficient of the compressive strength and the regain of the strength at longer time.

**Table 2:** Evolution of the SAI during the solidification process.

Ageing, d	S1	S3	S5
2	103.42	91.99	81.32
45	142.41	139.10	96.9

**Fig. 5:**
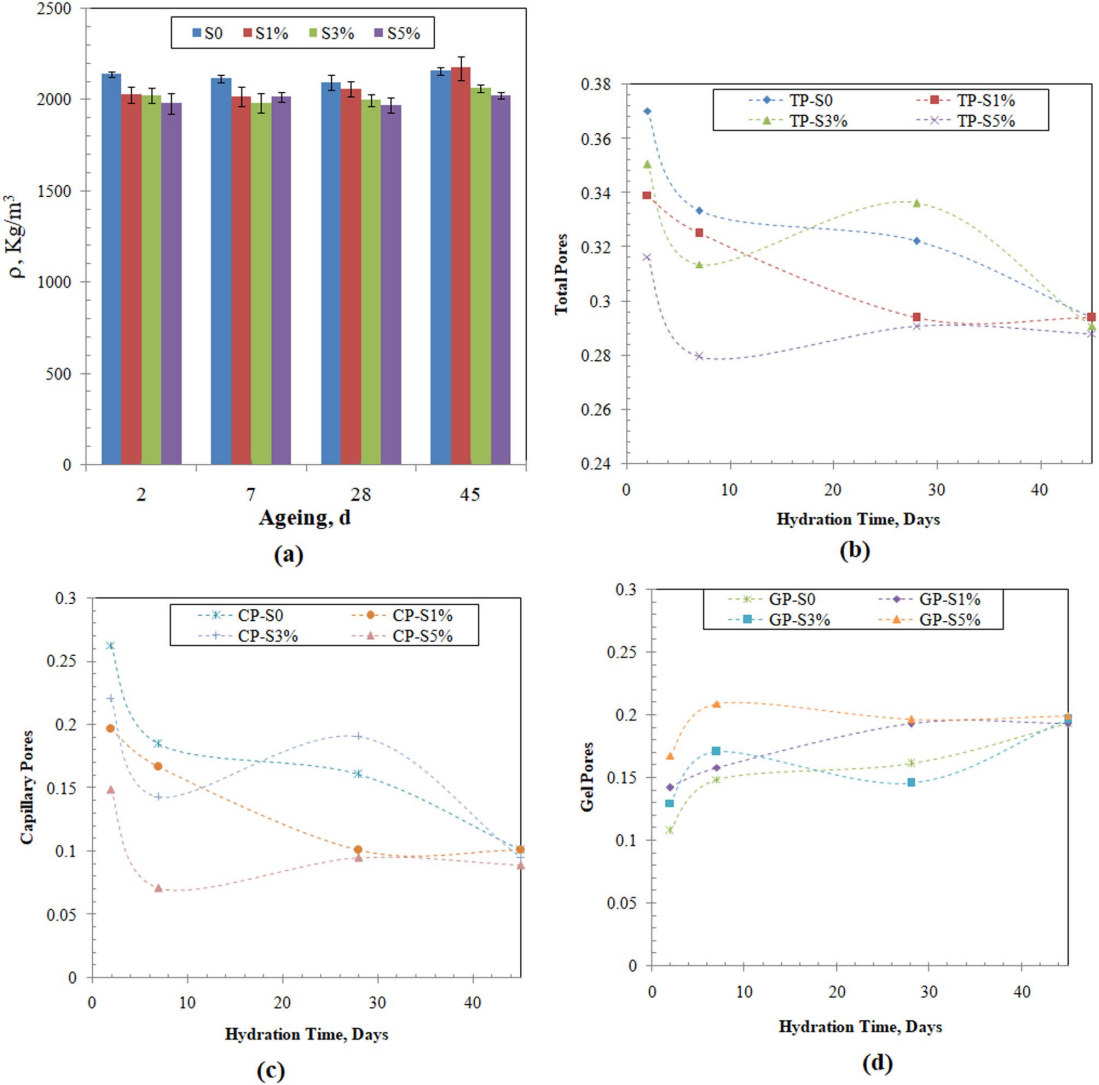
Evolution of the solidification process (**a**) density, (**b**)  total pores, (**c**) capillary pores, and (**d**)  gel pores.

The variation of the densities of the cementitious samples is fairly constant with small increases with time, which is attributed to the progress of the hydration reaction (Fig. 5a). The values of the total, capillary, and gel pores in the studied samples we calculated using (Eqs. 5–7), and the results are presented in (Fig. 5(b-d )). The evolution of the pore structures of the solidified cementitious materials is very much affected by the mix design of the sample, where the variation of the water to cement ratio, the nature and presence of fillers, and reactive supplementary materials can largely impact this evolution^45,81,88,89^. Total pores in the studied samples at age 45 equal 0.294, 0.294, 0.291, and 0.288 for samples S0, S1, S3, and S5, respectively. The calculated total pores are lower than that reported for modification of cement using micro-silica and suspension aid at w/c ratio 0.526^88^, and for cement composite containing 1% NS at water to cement ratio 0.29^89^, but higher than that of cement composite at water to cement ratio 0.5^81^ (Fig. 5b), illustrate the progressive reduction in the value of the total porosity with increasing the curing time, where the calculated porosity at 2 days are in the range 0.37–0.31 are reduced by 20.65, 13.36, 16.97, and 8.95% at days 45 for samples S0, S1, S3, and S5, respectively. This reduction can be attributed to the continuous filling of the capillary pores with the progress of the solidification process^45,54^. Moreover, the increase in the incorporation of the CNS in samples S3 and S5 lead to a slight reduction in the total pores. The evolution of the gel pores in the studied samples was evaluated by calculating the fraction of the gel pores to the total pores, and the results are listed in Table 3.

**Table 3:** Evolution of the gel pores during the solidification process.

Ageing, d	S0, %	S1, %	S3, %	S5, %
2	29.08	42.02	37.05	53.03
45	65.75	65.75	67.47	69.24

During the early solidification stage at 2 days, the modified samples have a higher gel fraction compared to that of the blank sample. This increased gel pores fraction can be related to the accelerating effect of the CNS on the reaction. This effect was reported for the use of micro-silica fume to modify cement composite in the presence of an accelerator at water to cement ratio of 0.5^82^. Yet it was not reported for the use of fly ash, micro-silica, and suspension aid at a water-to-cement ratio of 0.52, as the micro-silica contributed to the decrease of the macro-pores^88^. This confirms that as the mix design is changed, the pore structure and its evolution can change considerably. As the solidification process progresses, the gel pores continue to have an increased contribution to the total porosity, where the modified cementitious samples have higher gel pore fractions that are commensurate with the amount of CNS. This effect of the CNS on the increased fraction of gel pores might be attributed to its reaction with free lime to form amorphous calcium hydro-silicate and its reaction with portlandite to form denser CSH; thus, it reduces the loose and porous hexagonal plate-shaped portlandite^47,48,54^.

**Fig. 6:**
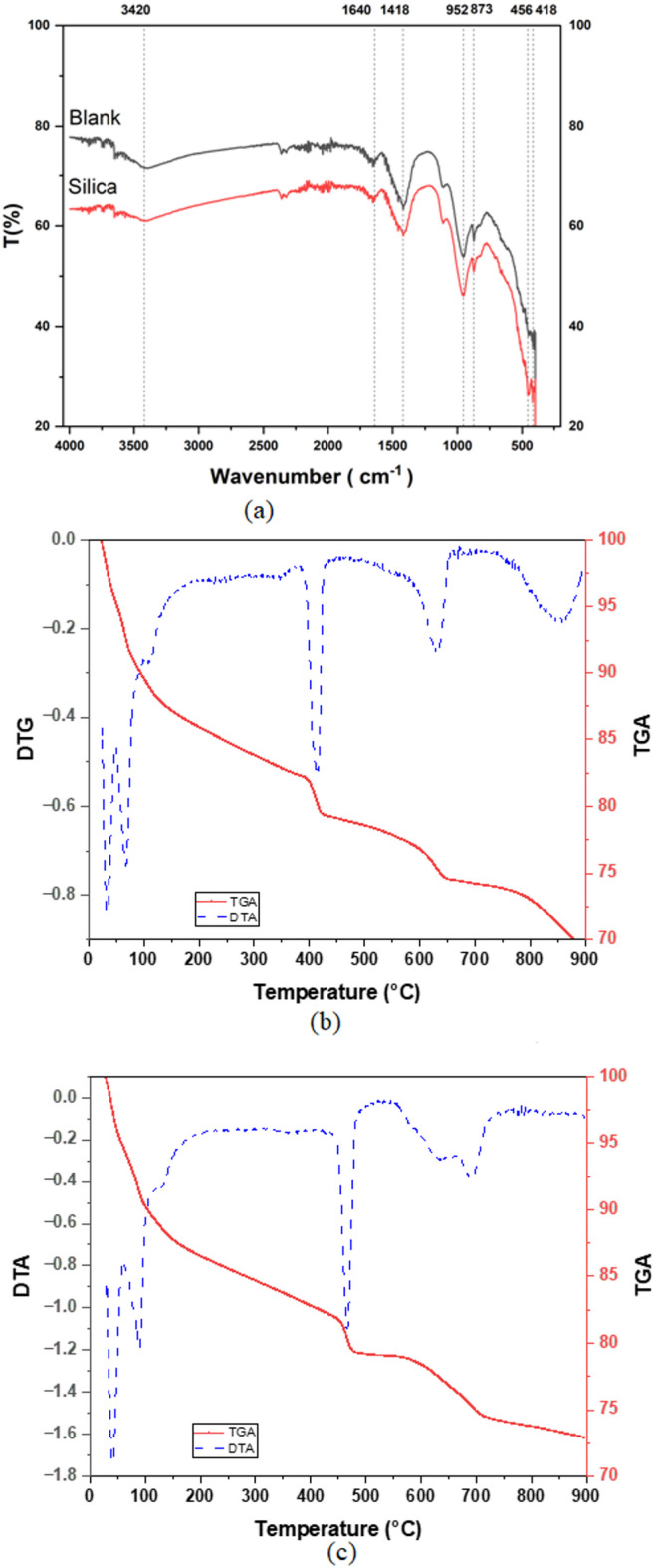
Role of CNS in the hydration phase development in cementitious materials (**a**) FTIR pattern for blank and CNS-supplemented sample (**b**, **c**) thermo-gravimetric data for blank and CNS-supplemented samples, respectively*.*

Figure 6a visualizes the differences in the formed function groups in blank and CNS supplemented samples at longer hydration ages. FTIR shows identical patterns for both samples except at low wavelength < 500 cm^-1^. In this regard, the O–H stretching and bending vibrations at 3420 and 1640 cm^-1^ are noted for both samples. The v_3_ and v_2_ vibrations of are detected around 1418 and 873 cm^-1^, indicating the progress of the carbonation process for the 9-month aged samples. The silicon tetrahedral (SiO_4_) characteristics peak in C-S–H/C-A-S–H hydration phases, i.e. Si–O-Si/Si–O-Al asymmetric stretching vibration (v_3_), appears 952 cm^-1^ which corresponds to the formation of C-S–H^49^. Weak in-plane Si–O bending vibrations *v*_2_ is detected at 456 cm^-1^. The larger intensity of *v*_*2*_ vibration is an indication on the larger amount of C-S–H in the supplemented cementitious material. This confirms the pozzolonic reaction of the CNS, where the enhanced formation of C-S–H gel due to the provision of extra reactive SiO₂ by CNS that consumes portlandite (Ca (OH)₂) and forms additional C-S–H. Moreover, small Si–O–Si asymmetric stretching band (~ 1118 cm⁻^1^) is detected, in the CNS-modified sample; the band is stronger and may be shifted a little to higher wave number compared with the Blank. These results imply a higher polymerization of silicate chains and denser C-S–H gel formation due to pozzolanic reaction of nano-silica, which confirms the increased contribution of the gel pores to the total porosity in the supplemented samples.

Figure 6b illustrates the TGA and DTG of blank cement sample, total weight loss is about 33% over the studied temperature range, I.E. 900 °C. These losses are attributed to the loss of weakly bonded water sorbed on the external surface, dehydration and dehydroxylation of the hydrated cement phases and de-carbonation of calcium carbonate phases^52^. These gradual losses correspond to three distinguished DTG peaks (40.34, 88.24, 467.39 °C) and a doublet (640, 689, 57 °C). They are ascribed to the release of weakly bounded water (5.171%), dehydration of the ettringite/AFT/CSH (10.07%), dehydroxylation of portlandite (5.676%), and the decomposition of the carbonate phases (6.109%). The presence of a diffused doublet carbonate peaks refers to the decomposition of distinguished carbonated phases, that include poor crystalline (shoulder below 600 °C), less crystalline phases related to the decomposition of the hemi/mono carbonates and calcium carbonate from the carbonation of Portlandite/ CSH and it is dominant in the range 600–650°C^90^. Finally, the decomposition of the carbonated lime and it is centered at 689.57 °C with no sign for any phase transformation above 800 °C. The thermo-gravimetric data of the CNS supplemented cementitious sample, showed fairly similar peaks at lower temperatures (Fig. 6c) ascribed to the release of weakly bounded water (4.71%), dehydration of the ettringite/AFT/CSH (10.42%), dehydroxylation of portlandite (6.337%), where the sorbed water on the material surfaces is lowered than that in the blank sample. In addition, the hydration products are slightly increased over that of the blank sample. The clear difference between both samples is related to the nature of the formed carbonation products and their respective amount and the presence of C-S–H of low Ca/Si ratio. In this context, the carbonation products in the CNS supplemented material are mainly of less crystalline hemi/mono carbonates and calcium carbonate from the carbonation of Portlandite/ CSH. This confirms that at this age no presence of free lime in the sample that are responsible for the formation of well crystalline phases, and indicate the better durability of the CNS supplemented sample compared to that of the blank sample. Moreover, the transformation of CSH into wollastonite is an indication of the presence of C-S–H- of low Ca/Si ratio that is formed from the pozzolanic reaction of the CNS^91^.

## Solidification mechanism

**Fig. 7:**
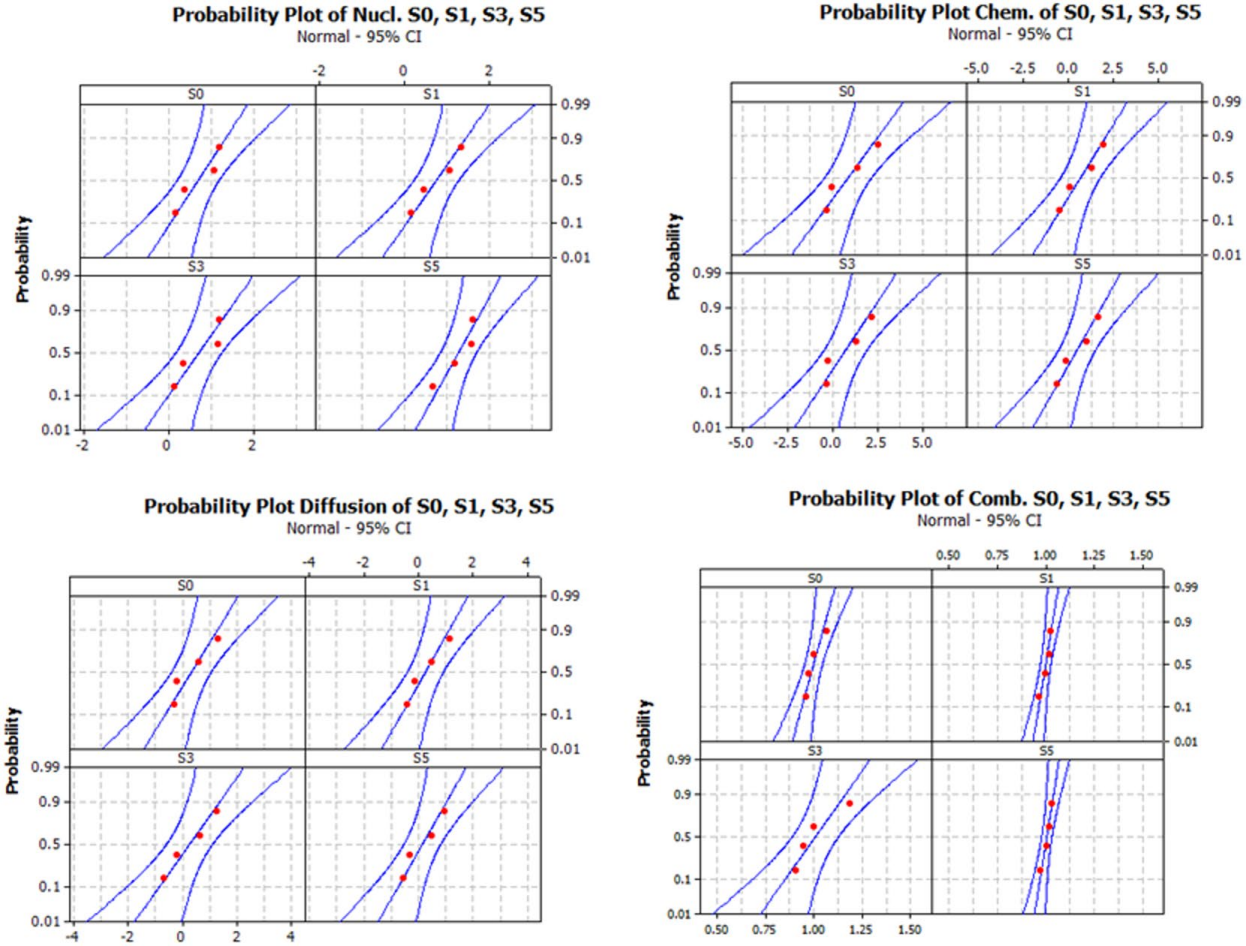
Statistical analysis of the regression error for the solidification mechanism investigations.

The nonlinear regression analysis of the experimental compressive strength data to the rate Eqs. (9–11) was conducted, and the regression errors were statistically analyzed to check their normality and homoscedasticity, as illustrated in Fig. 7 and in Table 4. The examination of the normal probability plots indicates that these errors follow a normal distribution without the presence of outliers and p-values are larger than the significant value, indicating that the errors follow normal distribution and homoscedastic.

**Table 4:** Identification of the solidification mechanism.

Model	S0	S1	S3	S5
Nucleation and Growth				
p-value	0.267	0.578	0.153	0.270
k_NG_	0.029 ± 0.020	0.038 ± 0.026	0.029 ± 0.020	0.223 ± 0.187
n	1	1	1	1
R^2^	0.901	0.979	0.471	0.0728
Diffusion				
p-value	0.348	0.653	0.677	0.505
k_D_	0.002 ± 0.001	0.003 ± 0.001	0.002 ± 0.001	0.005 ± 0.002
n	3	3	3	3
R^2^	0.949	0.984	0.666	0.577
Chemical reaction and mass transfer				
p-value	0.433	0.608	0.232	0.574
k_i_	0.007 ± 0.004	0.009 ± 0.004	0.007 ± 0.004	0.011 ± 0.005
n	3	3	3	3
R^2^	0.927	0.965	0.656	0.467
Combined nucleation and diffusion				
p-value	0.359	0.465	0.273	0.22
b	0.365 ± 0.134	0.418 ± 0.125	0.475 ± 0.121	0.641 ± 0.113
k_NG_	0.783 ± 0.615	1.61 ± 3.17	0.87 ± 0.559	0.887 ± 0.425
k_D_	3.93E-4 ± 5E-5	4.18E-4 ± 5E-4	7.82E-5 ± 2E-4	0 ± 2.47E-6
R^2^	0.974	0.979	0.742	0.949

**Fig. 8:**
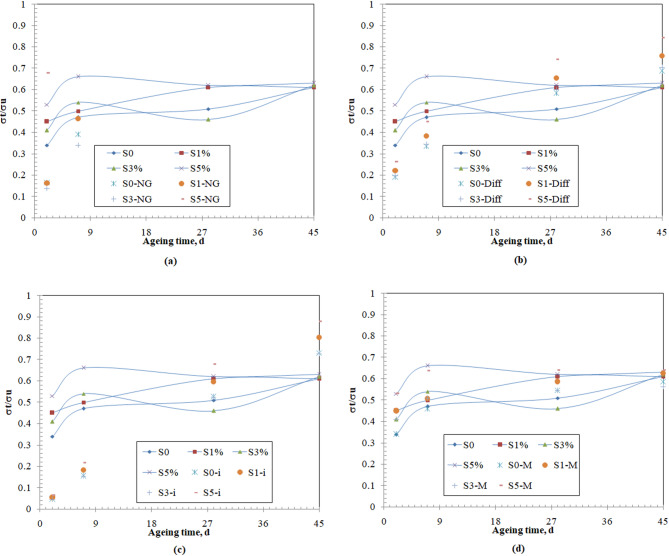
Solidification mechanisms (**a**) nucleation and growth, (**b**) diffusion, (**c**) chemical reaction and mass transfer, and (**d**) combined nucleation and diffusion.

The results of the regression are illustrated in Fig. 7 and Table 4. On one hand, the application of the nucleation and growth model to predict the solidification process is leading to considerable under estimation in the solidification performance measure during the first week of the process (Fig. 8a). On the other hand, the application of the diffusion model or the chemical reaction and mass transfer model will lead to similar underestimation of the solidification performance measure during the early week of different magnitudes and overestimation of this performance measure (Fig. 8b, c). In comparison with published data on the value of the exponents n, the predicted values are in conformance with published data^45,50,92–95^. The values of the correlation coefficient between the experimental data and the predicted data (R^2^), listed in Table 4, indicate that the diffusion model is the most appropriate model to predict the behavior of the solidification performance measure over the studied time.

The application of the combined nucleation and growth and solid-state diffusion and diffusion-controlled nucleation growth model (Fig. 8d) represents the experimental data very well throughout the studied ageing time, with the highest values for the correlation coefficients for all the studied samples. This reveals that the solidification process during the studied period is controlled by two simultaneous solidification mechanisms, namely nucleation and growth, and diffusion. The progressive incorporation of the colloidal nano-silica will increase the contribution of the nucleation and growth mechanism in the solidification process. In this respect, it was reported that the hydration of closely sized pure tri-calcium silicate is governed by the nucleation and growth mechanism in both acceleratory and post-acceleratory periods^50^. The presence of 4% C-S-H during the hydration of the tri-calcium silicate was concluded to have a catalytic effect on the nucleation of the hydration products^93^. In a more complex cementitious composite, which consists of cement and fine and coarse aggregates, it was reported that the presence of nano-silica contributes to enhancing the nucleation during the solidification process^47,48^. In an innovative cementitious system, comprising calcium sulfoaluminate cement, calcined phosph-gypsum, steel slag, and lime powder, it was found that the hydration is controlled by both nucleation and growth, and diffusion^95^. Hence, it could be concluded that the hydration of the cementitious material relies on the combined nucleation and growth, and diffusion mechanisms. The introduction of colloidal nano-silica into the system will shift the contribution of the nucleation and growth mechanism to the overall solidification process to higher values by increasing the CNS content, where 3% and 5% incorporation in the system leads to an increase in the nucleation rates and reduced the diffusion rates.

Based on the above results, CNS incorporation had both beneficial and adverse effects on the solidification performance of cementitious materials. On the favorable side, it significantly accelerated the solidification confirming its role as an effective hydration accelerator. This behavior stems from its high surface area and roughness, which provide additional nucleation sites for hydration products. It improved the soundness, particularly at 3% CNS, demonstrating its potential to enhance the solidified material stability by reacting with free lime to form calcium silicate hydrates. This result was confirmed by the absence of the carbonation peaks related to the lime carbonation in that sample (Fig. 6c). Moreover, the increased gel pore fraction with CNS incorporation indicates refinement of the pore structure, supporting denser hydration products that are critical for long-term durability. Importantly, mathematical sensitivity analysis revealed that low CNS dosages (1.5–3%) can positively influence compressive strength at later curing stages, suggesting that carefully optimized incorporation levels may offer a balance between strength retention and microstructural enhancement. The investigations of the aged samples, i.e. 9 months samples, confirmed the improved durability of the CNS supplemented samples at 3% dosage.

Conversely, higher CNS contents (≥ 5%) introduced challenges. Excessive water adsorption by nano-silica reduced the free water available for cement hydration, leading to diminished compressive strength over time. This strength reduction, coupled with minor expansion effects, underscores the non-monotonic behavior of CNS and highlights the need for dosage optimization. Additionally, while CNS promotes nucleation and growth mechanisms, excessive incorporation suppresses diffusion-driven hydration, potentially limiting long-term strength development.

Taken together, these findings demonstrate that CNS is not merely an additive but an active modifier of hydration pathways. At optimized dosages, it offers tangible advantages: faster setting, improved soundness, refined pore structure, and enhanced nucleation processes. However, its adverse effects at higher concentrations stress on the importance of controlled incorporation. This dual behavior provides new mechanistic insights into nano-modified cement solidification, offering pathways to tailor cement formulations for the stringent requirements of nuclear waste immobilization and disposal backfill.

Finally, in comparison with a similar study conducted on an innovative cementitious system composed of slag/fly ash-based geopolymers, in that study Luo et al. (2025) focused on how initial CaO/Al₂O₃ and SiO₂/Al₂O₃ ratios govern geopolymerization–hydration synergies and stabilization of waste clay. That study highlighted the importance of tailoring compositional parameters to optimize solidification performance. In the OPC-based matrix, CNS dosage critically influences nucleation/growth versus diffusion-driven hydration, whereas in the geopolymer system, oxide ratios dictate gel formation and long-term strength. Despite differences in binder chemistry, a common theme emerges—microstructural evolution, whether via refined gel pores in CNS-cement or polymeric network densification in geopolymers, directly controls macroscopic properties such as strength and dimensional stability. Benchmarking these findings underscores that both CNS modification and geopolymer design represent complementary strategies for advancing durable waste encapsulation matrices^96^.

## Conclusions

Nano-silica was prepared following the sol-gel method and was characterized to identify its chemical and physical characteristics. The prepared material is amorphous, containing about 20 wt.% water in the form of weakly bound water and chemically sorbed water. It has a particle size less than 2.49 nm, and a fairly fixed zeta potential equals -33.9 mV that confirms its ability to form colloidal solutions. The following conclusions were obtained from this investigation.

1. The incorporation of CNS in the cementitious material reduces the initial and final setting times, where a non-monotonic effect of the progressive increase of CNS in the cement samples is evident and was attributed to the availability of a larger surface area to adsorb the water in the samples.
2. CNS incorporation into the cementitious mix design up to 3% enhances the soundness of the solidified material due to the reduction of the LSF in the raw sample and the increased reaction of the silica with the lime.
3. CNS incorporation decreases in the compressive strength of the solidified samples over the examined curing time compared to that of the reference sample, but still has acceptable performance compared to the target value required for applications in radioactive waste immobilization or backfilling the radioactive waste disposal (10MPa).
4. Within the first week of the solidification reaction, the development of the strength is mainly due to the hydration of C_3_S and C_2_S. A small increase in the normalized sensitivity of the compressive strength is noted for the CNS supplemented samples and was attributed to the reaction of the nano-silica with the free lime as revealed from the Le Chatelier test.
5. Increasing CNS incorporation > 1% will lead to a loss in the strength at 28 days, due to the shrinkage associated with the presence of nano-silica; but the pozzolonic reaction of CNS will lead to a regain in the strength that depends in its magnitude with the amount of CNS.
6. The increased gel pores contribution to the total porosity with increasing the CNS incorporation was linked to CNS reaction with free lime to form amorphous calcium hydro-silicate and its reaction with portlandite to form denser CSH; thus, it reduces the loose and porous hexagonal plate-shaped portlandite.
7. The TGA/DTG and FTIR results confirmed the improved durability of the CNS-supplemented material over 9 months, where it allowed for the consumption of lime , eliminates its contribution to the carbonation, allowed for continuous formation of C-S–H of low ca/si ratio, and reduced the sorbed water on the sample surface.
8. The hydration of the cementitious material relies on the combined nucleation and growth, and diffusion mechanisms. The introduction of CNS into the system will shift the contribution of the nucleation and growth mechanism to the overall solidification process to higher values, where 3% and 5% CNS incorporation in the system leads to an increase in the nucleation rates and reduce the diffusion rates.

## Data Availability

The datasets generated during and/or analysed during the current study are available from the corresponding author on reasonable request.
